# Scaling up pediatric nurse specialist education in Ghana – a longitudinal, mixed methods evaluation

**DOI:** 10.1186/s12912-021-00550-1

**Published:** 2021-02-16

**Authors:** Roxana Salehi, Augustine Asamoah, Stephanie de Young, Hannah Acquah, Nikhil Agarwal, Sawdah Esaka Aryee, Bonnie Stevens, Stanley Zlotkin

**Affiliations:** 1 Centre for Global Child Health, Hospital for Sick Children, Toronto, Canada; 2 Ghana College of Nurses and Midwives, Accra, Ghana; 3 Lawrence S. Bloomberg Faculty of Nursing and Faculties of Medicine and Dentistry, University of Toronto, Toronto, Canada; 4 Departments of Pediatrics, Nutritional Sciences, Dalla Lana School of Public Health and the Munk School of Global Affairs and Public Policy, University of Toronto, Toronto, Canada

**Keywords:** Evaluation, Nursing education, Global Health, Ghana, Pediatrics, Nursing, Low middle income countries, Child mortality

## Abstract

**Background:**

Inadequate health human resources is a key challenge to advancing child survival in Ghana. Nurses are an essential human resource to target because they represent the largest portion of the health workforce. Building on lessons learned from our pilot pediatric nurse training project and World Health Organization guidelines for transforming and scaling up health professional education, this project aimed to; train 500 pediatric nurse specialists through a one-year training program; develop and integrate a critical mass of pediatric nursing faculty and establish a national standardized pediatric nursing curriculum. This study aimed to evaluate the effectiveness of a national pediatric nurse training program in Ghana at the end of 4 years, including eight cohorts with 330 graduates.

**Methods:**

This was a mixed-method evaluation with surveys, focus groups and a pre-test/post-test design. Before and after surveys were used to measure knowledge and confidence at baseline and graduation. Objective Structured Clinical Examinations (OSCE) were used to measure clinical skills at baseline, graduation, and 14 months follow-up. At the end of every module, surveys were used to measure students’ satisfaction. Focus groups at graduation qualitatively measured program outcomes. Repeat focus groups and surveys at 14 months after graduation captured the graduates’ career progress, experiences reintegrating into the health system and long-term program outcomes.

**Results:**

Overall, the graduates completed the program with significantly increased knowledge, confidence, and clinical skills. They also had increased job satisfaction and were able to apply what they learned to their jobs, including leadership skills and gender-sensitive care. Data from 14-month follow-up OSCEs showed that all graduates remained competent in communication, physical assessment, and emergency care, although some obtained a lower mark compared to their performance at graduation. This finding is linked with the observation that the amount of mentorship, support from leadership and equipment that the graduates accessed from their respective facilities varied.

**Conclusions:**

Mixed-methods evaluations demonstrated significant increases in knowledge confidence and skills by completing the program and maintenance of skills more than 1 year after graduation. Findings have implications for those working on the design, implementation, and evaluation of nursing education interventions in low- and middle-income countries.

**Supplementary Information:**

The online version contains supplementary material available at 10.1186/s12912-021-00550-1.

## Background

Globally, health professional education has not kept pace with health care challenges [1] which has limited the ability of health professionals to address the Sustainable Development Goals’ (SDG) agenda including reducing neonatal and under five mortality. Investment in nursing education in particular has not been commensurate with the need and spending on nurses’ and midwives’ education is disproportionately low compared to their representation in the global health workforce. Only one-quarter of the global expenditure on health worker education is spent on nursing and midwifery [2].

Underinvestment in nursing education has resulted in capacity constraints and challenges including a shortage of nursing faculty, outdated curricula that are mainly didactic “talk and chalk” rather than practice-based curricula, insufficient training in patient- and family-centred care, and shortage of teaching equipment [3, 4]. Training nurses to provide specialized, patient- and family-centred pediatric care is needed as evidence has shown increasing the density and specialization of nurses and midwives, who comprise over 50% of the global health workforce, results in improved health outcomes [5, 6].

Ghana has been investing in the training of pediatric nurses since 2010 in an effort to address health system shortcomings and reduce child mortality from preventable causes. A pilot pediatric nurse training program was established in 2010 and a 2013 evaluation of the pilot [7] indicated that the pediatric nurse graduates were applying advanced clinical competencies and assuming new roles and responsibilities as leaders in pediatric nursing care. Health system stakeholders recommended expanding the training to new sites across Ghana and increasing enrollment to meet national targets and to create a critical mass of pediatric nurses in the country. As such, the *SickKids-Ghana Pediatric Nursing Education Partnership* (PNEP) was established as an international effort to reduce child mortality in Ghana by scaling up pediatric nursing education across the country to further increase the concentration of highly skilled pediatric nurses who are critical to improving health outcomes for children [8, 9]. PNEP was established as a capacity building development initiative between the Hospital for Sick Children (SickKids) in Toronto, Canada and the Ghana College of Nurses & Midwives, Ghana Health Service and the Ghana Ministry of Health (MOH) in support of the MOH’s goal to train 1500 pediatric nurses by 2025.

Through PNEP, a one-year post-basic, practice-focused pediatric nursing education program was established at three training sites in Ghana with a target of training 500 pediatric nurses by September 2020. PNEP partners worked to ensure equitable recruitment from underserved areas into the program, increased recognition and remuneration for nurses upon graduation, and effective deployment and retention in the Ghanaian health care system. With an additional focus on positioning nurses as leaders who can not only improve patient outcomes but also advocate for gender and health equality and closing the global gap in health leadership positions held by women [2, 10], PNEP’s goals are aligned with advancing SDGs #3 (*Good Health and Wellbeing and its target of achieving universal health coverage),* #4 *(Quality education),* and #5 (*Gender equality)*.

A rigorous monitoring and evaluation plan was established at the onset of PNEP to support continuous learning and improvement and to ensure quality education standards were met. This manuscript presents the evaluation of PNEP’s one-year pediatric nursing education program in Ghana and focuses on program outcomes (confidence, knowledge, clinical skills) at graduation and 14 months after graduation; reintegration to the Ghanaian health system 14 months after graduation; and overall satisfaction with the program.

### Pediatric associate program

PNEP’s one-year pediatric nursing education program, hereafter referred to as ‘the program”, is delivered by the Ghana College of Nurses and Midwives, hereafter referred to as ‘the College’. Nurse educators from SickKids and Ghanaian nursing faculty collaborated to develop a national, accredited, competency-based curriculum that would be taught at all program sites. Program curriculum focuses on foundational pediatric nursing principles such as family centred care, strengths-based nursing, and gender equality, the provision of primary care and community care, managing acute and changing conditions in hospital and emergency settings, and nursing leadership development.

To be admitted into the program, students must already be registered nurses of good standing with the Nursing and Midwifery Council of Ghana, hold a minimum of a diploma certificate in nursing, and have at least 3 years of work experience. Delivery of the program includes interactive lectures, case-based learning, simulation, and experiential learning through an intensive clinical practicum to ensure integration of theory with practice. Students spend 30% of their time in class and the skills lab and 70% of their time in selected facilities at training centres for the clinical practicum.

**Fig. 1:**
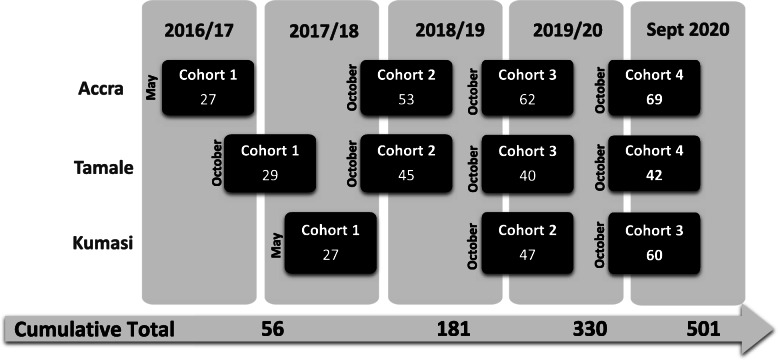
PNEP stepwise approach to class sizes by cohort and training site from 2016 to 2020. Cumulative totals for each year are shown at the bottom, leading to 501 students who have been trained over the duration of PNEP

To support country-wide recruitment into the program, the country was divided into three zones (southern, middle and northern zones) with Accra (capital city of the Greater Accra Region and national capital), Kumasi (capital town of the Ashanti region), and Tamale (capital city of the Northern region) serving as the regional training centres. Classrooms and Skills Labs were established at each training centre. PNEP employed a step-wise approach to recruitment of cohorts of students at each training centre over 5 years with the cumulative total of 501 students (Fig. 1). The recruitment strategy aimed to recruit nurses mainly from district level hospitals, with particular focus on underserved regions, as identified by Ghana Health Service.

## Methods

### Evaluation Framework & Methodology

The Monitoring & Evaluation (M&E) Plan for PNEP was a mixed methods, qualitative/quantitative design with concurrent data collection and analysis that was developed in consultation with key stakeholders and received approval through the SickKids Quality Improvement Committee in May 2016 and from the Ghana Health Service Ethics Review Committee in August 2016. PNEP’s M&E plan is built on the notion that evaluation of nursing education programs is a process that starts during education design and continues through delivery of the program, graduation of students and when graduates have gone back to work within the health system [11–14]. Specifically, the M&E plan had the following objectives:

1. Measure graduates’ program outcomes (confidence, knowledge, clinical skills) at graduation and 14 months after graduation;
2. Assess graduates’ reintegration to the Ghanaian health system 14 months after graduation; and
3. Examine if graduates were satisfied with the program and uncover areas for improvement.

This study focuses on the sections of the larger evaluation plan related to cohort data and the graduates’ perception of the program’s effectiveness. Appendix 1 provides a visual summary of the mixed methods design methodology. Qualitative and quantitative tools were used to collect, analyze and triangulate data and to share results in real-time for continuous quality improvement [11]. Three program outcomes (knowledge, confidence, clinical skills) were quantitatively and qualitatively measured (objective 1). Two of these outcomes - knowledge and confidence - were measured at baseline and at graduation. Clinical competence was measured at baseline, graduation and again 14 months after graduation (section 3.1 lists the cohorts who participated in each point in time). To capture graduates’ reintegration to the Ghanaian health system (objective 2), all graduates were invited to fill out a survey 14 months after graduation, and a selected number also participated in focus groups. To measure students’ satisfaction with the program and uncover areas for improvement (objective 3), students completed course evaluation surveys after each program module, and focus groups were held with each cohort at graduation.

### Evaluation tools

#### Multiple choice exam

A 21-question multiple-choice exam, covering the key content of the program curriculum, was used to measure nurses’ knowledge before they started the program, and again at graduation [13, 15].

#### Confidence questionnaire

A seven-item questionnaire was used to measure nurses’ confidence in seven key areas related to clinical practice before they started the program, and again at graduation [13, 15]. A five-point Likert-type scale was used, where one represented “not confident at all” and five represented “fully confident.”

#### Objective structured clinical examinations

Objective Structured Clinical Examinations (OSCEs), considered the gold standard for assessment of clinical competence [16, 17], were used to measure students’ clinical competence. During an OSCE, examiners present a standardized case to students and ask them to “think out loud”, and explain, demonstrate or simulate the steps they would take to provide patient care.

Clinical competence was measured at graduation and 14 months after graduation, using three OSCE stations to evaluate students in key areas required for every patient encounter:

1. Physical Assessment
2. Communication
3. Emergency: Assessing and Responding to a Deteriorating Patient

Fourteen months after graduation was chosen (instead of a longer lag time) due to the time limitations of the PNEP project period. Similarly, a baseline OSCE was conducted with only one cohort (Kumasi Cohort 2), due to time and resource constraints within PNEP. Prior to the baseline assessment, students were given an introduction to the structure of an OSCE since many students had not encountered this type of exam in their previous training. The baseline OSCE covered Physical Assessment and Communication stations. Competencies in Emergency Management were considered more advanced and not assessed at baseline.

All OSCEs were marked using a five-point scale adopted from Bondy’s validated scale [18]. The scale categories were:

1 = Not competent.

2 = Towards competent.

3 = Competent.

4 = Towards proficient.

5 = Proficient.

Detailed scale definitions are presented in Appendix 2. To pass an OSCE, students were required to score a three (Competent) or higher. The details of the development, piloting, and evaluation of OSCE training and tools are discussed in a forthcoming publication.

#### Focus groups

Seven to eight individuals from seven respective cohorts were invited to participate in in-person facilitated focus groups when they finished the program (for a total of seven focus groups and 55 participants). Informed written consent was obtained from all participants for all focus groups and facilitators were hired to run the focus groups with standardized questionnaires that were developed specifically for these focus groups. The graduates were asked to comment on the quality of the curriculum content, the teaching format, and ideas for improving the program. Maximum variation sampling was used [19] using the following criteria to capture variations in experiences:

- The region where the nurses worked before entering the program;
- The type of institution where they worked (e.g. district, teaching or regional hospital); and
- The number of years of experience as a nurse.

Focus group facilitators were external to the College. Focus group data were recorded, transcribed, and coded by two researchers. Codes were further analyzed using a thematic analysis approach [20]. Refreshments and a small honorarium (phone card) were provided for participation.

Fourteen months after graduation, three additional respective focus groups were conducted with a total of 38 graduates. Graduates were asked to describe their experiences of going back to the workforce and to discuss their challenges and achievements. Facilitators of these focus groups were staff at the College with little or no prior contact with the graduates. The same sampling strategy as above was used.

#### Follow-up survey

A follow-up survey was sent to all graduates 14 months after they finished the program in order to track retention within the Ghanaian health system and understand their career trajectory. Of particular interest was whether the graduates remained in their respective health facilities and whether they were promoted to a higher nursing rank with increased salary (as this was expected as part of the recognition and remuneration strategy employed by the Ghana Health Service for those who completed the program).

#### Course evaluation surveys

Graduates’ satisfaction with the program and areas for improvement were measured using course evaluation surveys throughout the year. For this study, the focus will be on *Overall Satisfaction*, measured using a five-point scale for both in-class modules and clinical practicums.

## Results

All statistical analysis was performed using IBM SPSS Statistics, version 26. Descriptive statistics were conducted to summarize the demographic characteristics and the 14 months follow-up survey data and to calculate the level of confidence, knowledge, and clinical skills (OSCE station mean scores). Wilcoxon Signed Ranks for paired samples was used to measure changes in both knowledge (continuous variable, not normally distributed) and confidence (scale variable from one to five) before and after the program. Focus group data were recorded, transcribed, and coded by two researchers. Codes were further analyzed using a thematic analysis approach [20].

The analysis is presented as aggregate data. Cohort-specific analysis, however, has been conducted throughout, to ensure that quality of education was consistent across training sites and over time.

### Data inclusions and exclusions

This study focuses on data for the eight cohorts that have so far graduated from the program. Appendix 1 provides a visual summary of data inclusions and exclusions. Demographic data are complete for all eight cohorts. Pre-post analysis and OSCE results from Accra Cohort 1 are not included here because, at the time the data was gathered, the program was getting started, tools were being piloted, and ethics approval was underway. Focus groups at graduation were conducted with all cohorts except Kumasi Cohort 2, due to project constraints. Fourteen months follow-up OSCEs and focus groups were conducted with Accra and Tamale Cohort 2 and Kumasi Cohort 1, but not conducted with Accra Cohort 1 and Tamale Cohort 1, due to resource and timeline constraints. At the time of writing, 14 months had not yet passed since the Accra and Tamale Cohort 3 and Kumasi Cohort 2 graduated, and hence, they had not received the follow-up survey. Similarly, post-program data (i.e. results at graduation and at 14 months after graduation) was not available for the cohorts that graduated in September 2020 (Accra Cohort 4, Tamale Cohort 4, and Kumasi Cohort 3). Nine students completed an accelerated version of this program (discussed in another forthcoming manuscript). These nine students participated in OSCEs at graduation but did not participate in focus groups or other assessments.

### Description of students

Table 1 shows the key demographics at enrollment of the eight cohorts under discussion. Aggregate cohort size is 330 students (*N* = 330) across the Accra, Tamale and Kumasi training centres and there were no missing responses. On average, students had 6 years of work experience and were in their early 30s. The range was large for both age and years of experience; this shows that the program attracted both novice and experienced nurses. More than half entered the program with a diploma certificate, as this is the minimum entry requirement for the program at the College. The majority were from district hospitals. Students were recruited from all 16 regions of Ghana, with half being from regions identified as underserved.

**Table 1:** Characteristics of first eight cohorts of students at enrollment (2015 to 2018)

Characteristic	N (%)
Age in years, Mean (SD)	31.3 ± 3.7
Years of experience as a nurse **Mean (SD)**	5.8 ± 3.5
Female	250 (76)
Male	80 (24)
**Highest level of education**	
Diploma	211 (64)
BSc	105 (32)
Masters	11 (3)
Others^a^	3 (1)
**Type of facility**	
Teaching Hospital	23 (7)
Regional Hospital	37 (11)
District Hospital	227 (69)
Polyclinic	11 (3)
Health Centre	19 (6)
CHPS^b^	2 (1)
Nursing training school	11 (3)
Recruited from underserved regions	181 (55)
**Rank**	
Principal Nursing Officer	4 (1)
Senior Nursing Officer	37 (11)
Nursing Officer	36 (11)
Senior Staff Nurse	178 (54)
Senior Staff Midwife	7 (2)
Staff Nurse	56 (17)
Staff Midwife	2 (1)
Other^c^	10 (3)

^a^One student had a PhD, another had Bachelor of Arts and another student had Bachelor in Education in Health Science

^b^*CHPS* community-based health planning and services

^c^“Other category” consisted of one deputy director of nursing service and nine nurse educators from the Nursing Training Colleges in Ghana

### Program outcomes

#### Knowledge

The Wilcoxon signed-rank test showed that the mean knowledge test score increased from 52% ±11.2 at baseline to 71% ±9.2 at graduation, a 37% increase, which is statistically significant (Z = -14.3, *n* = 293, *P* = 0.000). Variations existed within and between cohorts in terms of baseline knowledge (values not shown); however, across all cohorts, graduates had increased knowledge compared to baseline and the change was statistically significant.

#### Confidence

Changes in confidence was assessed for seven cohorts of students between baseline and graduation through a 5-point Likert scale survey (5 meaning fully confident). Results in Table 2 show that in all domains of practice, students reported increased confidence after completing the program. The biggest change in confidence was for physical assessment and history taking. The aggregate results below were similar to cohort-specific analysis (not shown). Focus group discussions (Tables 5 and 6) further validated these results.

**Table 2:** Change in confidence for seven cohorts of students from baseline to graduation (*n* = 293^a^)

	Confidence		
Domains of practice	Mean (SD) at baseline *n* = 293	Mean (SD) at graduation *n* = 293	P^b^
History taking	2.6 (0.9)	4.7 (0.5)	0.00
Physical Assessment	2.5 (0.9)	4.7 (0.5)	0.00
Communication with physician	3.0 (0.9)	4.6 (0.6)	0.00
Dealing with pediatric emergencies	2.9 (0.9)	4.3 (0.6)	0.00
Managing conflict at the workplace	3.6 (0.9)	4.3 (0.7)	0.00
Presenting a patient to a physician	3.1 (0.9)	4.6 (0.6)	0.00
Alerting a colleague of critical issues	3.8 (0.9)	4.8 (0.5)	0.00

^a^The missing data include Accra Cohort 1 and those who completed the accelerated certification

^b^Wilcoxon Signed Ranks Test for Paired Samples

#### Clinical competence and skills

Tables 3 and 4 summarize the results of OSCEs which measure clinical competence. Table 3 compares OSCE station means (out of 5) at graduation vs. 14 months-follow-up for three cohorts (Kumasi Cohort 1, Tamale and Accra Cohort 2). Complete data were available for 74 individuals as 15% did not attend the 14 months follow-up. The results in Table 3 show that graduates retained their competencies in key areas of pediatric care over time as OSCE station means remained above 3 (the passing grade), but that station means for Communication and Emergency OSCEs were lower at 14 months follow-up compared to graduation. The station mean for Physical Assessment OSCE was unchanged.

**Table 3:** Comparison of OSCE station means at graduation vs. 14 months follow-up

OSCE Station	Graduation (*n* = 74) Mean (SD)	Follow-up (*n* = 74) Mean (SD)	P^a^
Physical Assessment	3.8 ± 0.5	3.7 ± 0.4	0.1
Communication	4.2 ± 0.4	3.5 ± 0.4	0.000
Emergency	4.2 ± 0.3	3.4 ± 0.6	0.000

^a^Wilcoxon Signed Ranks Test for Paired Samples

**Table 4:** Distribution of competencies for students at baseline, graduation and 14 months follow-up OSCEs

	Physical Assessment			Communication			Emergency		
Level of competency	Baseline (*n* = 44)	Graduation (*n* = 74)	Follow-up (*n* = 74)	Baseline (*n* = 44)	Graduation (*n* = 74)	Follow-up (*n* = 74)	Baseline (*n* = 44)	Graduation (*n* = 74)	Follow-up (*n* = 74)
1. Not competent	9%	0%	0%	11%	0%	0%	Did not conduct	0%	3%
2. Towards competent	57%	4%	1%	41%	1%	8%		0%	10%
3. Competent	32%	51%	65%	48%	23%	74%		19%	70%
4. Towards proficient	2%	45%	32%	0%	72%	18%		72%	18%
5. Proficient	0%	0%	1%	0%	4%	0%		10%	0%

Percentages are rounded so they may not add up to exactly 100%

Table 4 provides further details about changes in competencies over time by delineating the distribution of competencies among students and graduates at baseline, graduation, and 14 months follow-up. The baseline was Kumasi Cohort 2, comprised of 44 individuals (three individuals did not attend the baseline OSCE). Graduation and 14 months follow-up cohorts were Kumasi Cohort 1, Accra Cohort 2, Tamale Cohort 2 and, for these cohorts, complete data was available for 74 individuals. Of those who were eligible to participate in the follow-up OSCEs, 15% (14 out of 88) did not attend. Table 4 shows that for all OSCEs, the majority of the students were not competent in the tested skills at baseline (66% in physical assessment and 52% in communication). At graduation, the large majority of students were either competent, towards proficient, or proficient (96% in physical assessment, 99% in communication, and 100% in emergency). The 14 months follow-up results show that the graduates have maintained their competencies but some who scored as ‘towards proficient’ or ‘proficient’ at graduation had declined to a lower category (competent, or towards competent). During Emergency OSCE at 14 months follow-up, 13% scored lower than the threshold (3 = competent), while that number for Communication OSCE was 8%, and for Physical Assessment OSCE it was only 1%.

A thematic analysis was conducted (Table 5) of seven respective focus groups that were held at graduation with a cumulative sum of 55 graduates (characteristics of the 55 graduates are outlined in Appendix 3). The analysis showed that students emphasized their increased leadership abilities and a more positive attitude towards their patients as some of the most important outcomes of the program. They also said they are better able to provide gender-sensitive care. Table 6 outlines the thematic analysis from three respective focus group discussions with 38 graduates 14 months after graduation (characteristics of the 38 focus group participants are outlined in Appendix 4). At 14 months follow-up, graduates gave concrete examples about how they are applying their leadership skills to affect change in their facilities. All graduates reported high levels of job satisfaction at 14 months follow-up due to their newly acquired clinical and leadership competencies. Their perception was that their skills are ultimately improving care for patients.

**Table 5:** Thematic analysis from focus group discussion with students at graduation

Category	Sub-Categories	Selected quotes
Satisfaction with the program	• Quality of teaching • Evidence-based content (curriculum) • Practical nature of the program	“*[T] he fact that faculty used different modalities to teach us, […*] *was very remarkable because this is the first time I have actually seen such things. They use videos, pictures to demonstrate to us. I think it was very good and helped us a lot.”* *“The most important thing I liked about the program was about the simulations, the objective structured clinical exam (OSCE) that we do,,. It makes you to retain something – the skills that you have learned […] After every module you have to go through OSCE to demonstrate what you have learned.”*
Challenges with the program	• Scheduling and intensity of the training within a one-year time frame	*“Sometimes, even more than 100 slides. And you are to write exams within six weeks. […*.] *The one-year program is too packed so if they can extend the time [that would be an improvement].”*
Knowledge, Skill & Attitude Outcomes	• Increased confidence in their decision-making abilities • Increased skills particularly in Physical Assessment • Effective communication with patients and families • More positive attitude towards their patients • Providing gender-sensitive care • Challenging gender norms	*“Before GCNM, I wasn’t doing most of the things […] I’m like, what am I using a stethoscope for? ... But when I came to GCNM, I realized the nurse can really use the stethoscope and it will really help her to get all the assessment findings that she wants.”* *“We were resuscitating a baby and the doctor was doing it. I realized that she was doing it the wrong way. Formerly, I’d have just stood there because she is the doctor, but now, I approached her and said no, we go about it this way.”* *“Coming through this program, I’ve realized that I have to have a good tolerance level with my patients, being able to explain to them even if the whole ward is saying they are very difficult.”* *“On the ward, when we are calling a parent we say, ‘mothers please come’ but we forget there may be a father amongst them. Or if the mother has stepped out and it’s her husband who is caring for the child and you say that mothers should come, then you are excluding the men. We have to change our language”*
Suggestions for program improvement	• Reduce fees to increase accessibility	*“One thing that prevents people from coming for the program is the high cost of tuition, if something can be done about it, and also the need to get study leave … a very big issue.”*

**Table 6:** Thematic analysis from focus group discussion with graduates 14 months after graduation

Category	Sub-Categories	Selected quotes
Program long term outcomes	• Increased job satisfaction • Improved leadership abilities to initiate change • Improved patient outcomes because of newly gained competencies	“*A boy came and the Physical Assessment findings and presenting complaints did not match. I assessed and suspected nephrotic syndrome. In the lab request, I added a urine [test]. The doctor came and asked what prompted me to check because the nurses he worked with never did that … [He said] ‘You have done well because if we had missed this, the child could have died.’ I felt satisfied*.” “*I organized a ward meeting with my staff. I have taught them emergency management, pain, NG tube. … I gave them the rationale and now they can practice in my absence*.” “*Before PNEP, I was at the OPD. There were cases we missed because we did not have the skills, some would have to die. But since I went back now, we manage it.”* “*Job satisfaction is 100%: to go home happy, to save a life, mothers see you in town and they come to greet you. Community members give you more respect*.”
Factors that support their reintegration to the health system	• Supportive management • Having another graduate from the program work in the same facility	*“My manager is a [graduate] herself, so she understands and supports me.”*
Challenges	• Lack of understanding of the pediatric nurse role • Lack of recognition in absence of a degree • Lack of equipment/infrastructure	“*People expect a degree BSc. They don’t understand the specialization.”* *“We have issues with logistics, you are trying to improvise. We don’t have pediatric nasal prongs, so after you use for one baby you have to reuse on other babies. NG tubes as well […] –you won’t have the right size*.”

### Reintegration within the Ghanaian health system 14 months after graduation

Of the 125 students eligible to take part in a 14 months follow-up survey, 124 participated (one graduate could not be reached). Of the 124 participants, all had remained in the Ghanaian health system with 119 being in same facility as at baseline, and five having moved to a different hospital. Of the 124 participants, 120 were promoted to Nursing Officer rank, an indicator of recognition and increased remuneration. Three were not promoted as they were already Nursing Officers at baseline, and one participant could not be promoted due to protocols in the military hospital where the graduate worked.

As shown in Table 6, supportive management and the presence of another graduate in the same facility were cited as key factors for their success, while a lack of management support, lack of infrastructure (equipment and space), and lack of understanding of their role were cited as challenges.

### Satisfaction with the program and areas for improvement

For the in-class portion of the program, the mean Overall Satisfaction score for all cohorts on a five-point scale was 4.35 ± 0.18 (*n* = 330). Overall satisfaction ranged between 4.0 and 4.6 among cohorts. For clinical practicum, satisfaction ranged from 2.9–4.9. Due to the varied nature of the facilities in which the students completed their practicum, an overall mean satisfaction score is not reported.

Table 5 summarizes the main points from the focus group discussions at graduation. The quality of the program in terms of teaching and content was rated very positively overall in focus groups at graduation. The program’s tight schedule, on the other hand, was cited by students as a challenge. They also suggested that reduced tuition fees would make the program more accessible to future candidates. Students wanted a degree instead of a certificate.

## Discussion

Overall, the results showed that the desired end-of-program outcomes were being met, and for the most part maintained over time. In addition, graduates were able to enhance their roles, careers and the care they provide by applying what they have learned in the program to their jobs and retained this knowledge well after the program ended. The program has also been successful in enhancing graduates’ careers through increased recognition (rank and salary), job satisfaction and by graduates advocating for a clearly defined pediatric nurse role in their health care setting. Despite areas for improvement, and system-level challenges (e.g. lack of equipment or hospital management support) outside the control of the program, the mixed methods evaluation demonstrates that the program was successful in creating a critical mass of highly skilled, knowledgeable, competent and confident pediatric nurse leaders.

In the program, graduates had statistically significant increases in knowledge and confidence and focus group discussions confirmed these quantitative findings. The largest change in confidence was related to physical assessment and history taking, which was notable because all focus group participants identified these two as areas they wanted to improve on.

At graduation, focus group participants reported a more positive attitude towards their patients and talked about having learned valuable leadership skills and competencies to provide gender-sensitive care. Subsequently, at 14 months follow-up, they gave concrete examples of how they applied those skills in their practice, including teaching their colleagues new skills and advocating for their patients to improve patient outcomes. All graduates reported high levels of job satisfaction at 14 months follow-up due to their newly acquired competencies and self-perception as being leaders who can make positive changes happen.

The significance of these results becomes more pronounced within the current context of health care provision in African countries. Disrespectful care and maltreatment of mothers (the primary caregivers) and patients as well as stigmatization of adolescents seeking health care services by health workers has been a known but largely ignored problem in many African countries, including Ghana [21–23]. Female children and women who care for them, especially those from rural and lower socioeconomic communities that have limited access to information and less power to make decisions about their health, are most vulnerable to poor treatment. The provision of gender-sensitive care by pediatric nurses in the Ghanaian context has the potential to remediate against a systemic barrier to the provision of high-quality, patient-and family-centred care.

Research has shown that transition of nurses from ‘competent’ to ‘proficient’ depends on a range of interconnected factors, including having the opportunity to practice the learned skills, receiving ongoing mentorship from their colleagues, a supportive work environment and an organizational culture that is open to change [14, 24–28]. In the early days of PNEP’s operations, stakeholders in Ghana commented that not all program graduates would be able to achieve their true potential once they returned to work due to some facilities’ limited capacity to support them and because the role of a pediatric nurse had not been clearly defined in Ghana. Results from this study were consistent with these comments as graduates noted this during focus groups at 14 months follow-up. Completing the one-year program resulted in the acquisition of the clinical competencies necessary to provide high-quality pediatric care in nearly all students at graduation, but 14 months later, although they had retained their competencies, their scores had declined compared to those at graduation for emergency management and communication OSCE. Some graduates said they do not get a chance to practice certain skills, such as emergency management, because of where they are placed within the hospital. Many cited lack of access to equipment, medications, and dedicated space for treating pediatric patients as impediments to their work.

A series of clinical observations and stakeholder interviews at clinical facilities where graduates are practicing are being planned for a future date to better understand how the results of this educational program influence organizational improvements in hospitals across Ghana and what strategies could be developed to support the graduates so that they can contribute to decreasing under-5 mortality in Ghana. One area where institutions like the College could exert direct influence in supporting graduates is through ongoing professional development opportunities via short courses and networking opportunities. In the case of PNEP, 14 months follow-up OSCEs were structured to not only complete the evaluation but also facilitate professional development sessions and debriefing with the faculty and graduates. The College is now in the process of expanding the number of professional development courses available.

Students’ overall satisfaction with the program was high, evident both from the overall satisfaction score for all eight cohorts and from focus group data. While the satisfaction rate for the in-class component of the program was consistently high among all cohorts, satisfaction with the clinical practicum varied slightly, likely due to variations in size and scope of clinical sites as well as number of preceptors available to mentor the students. The program team closely monitored satisfaction rates at each clinical site and made changes to the list of sites chosen as practicum sites as needed. One area for improvement revealed by focus group participants was the ratio of content to the time allocated to each module. Students consistently felt that the intensity and rigour of the program was more consistent with a first-degree program than a post-basic program. This feedback, along with other stakeholder consultations, informed continuous revisions to the program in each academic year.

### Strengths & Limitations

Study strengths include evaluating the longer-term outcomes of the program (at 14 months) which will offer stakeholders and perhaps other groups doing similar work, a broader perspective on the extent to which this type of program may strengthen the nursing workforce. Other strengths include the triangulation of designs and data types (qualitative and quantitative) for more robust conclusions and sharing the lessons learned in real-time for continuous quality improvement.

There are some limitations associated with this evaluation. Evidence shows that OSCEs with a larger number of stations tend to have higher reliability [17]. In the case of PNEP however, designing and running more than three stations was not possible because of limited clinical equipment and, more importantly, a limited pool of candidates that could be trained as raters. Rather than increasing the number of stations, priority was given to training the raters and standardized patients, to ensure consistency and reliability. Before baseline OSCEs, students were given an orientation to this exam format. Despite this training, the OSCE scores at baseline could have been higher if the students had prior experience with OSCE baseline [15, 29]. Since all cohorts were similar in terms of their key demographics (age, years of experience, type of facility where they work), the single baseline cohort was deemed appropriate to act as a reasonable comparison to other cohorts. It would have been preferable, however, if baseline data had been available for all cohorts.

Another limitation is that at the time of writing, results for eight of 11 cohorts had been included due to the timing of PNEP’s project period which ended in September 2020. Three cohorts graduated in September 2020 and a 14-months follow-up (November 2021) would only be possible well after PNEP ended. Given the patterns to date with the first eight cohorts it can be inferred that graduates will demonstrate similar results however further analysis after all 11 cohorts are completed would be necessary to validate this.

## Conclusions

PNEP’s project period ended in September 2020, however the program will continue forward under the operational leadership of the College. Although operating the program independently of PNEP presents new challenges for the College, the program has been setup to be sustainable in a manner that can continue to build the knowledge, confidence and skills of pediatric nurses. Continuing investment in education at the post-graduate level is required to develop nursing leaders in clinical, education and policy roles who can affect systems-level changes that ultimately benefit patients and in PNEP’s case - children. This evaluation has generated evidence regarding the benefits that a high-quality nursing education program can offer to nurses, the children they treat, and national health systems. Through high quality education, the program will continue to contribute to the ultimate outcome of reducing under-5 mortality and advancing multiple Sustainable Development Goals.

### Supplementary Information

**Additional file 1.**

**Additional file 2.**

## Data Availability

The datasets generated and/or analysed during the current study are not publicly available due to the data being proprietary and confidential records of the Ghana College of Nurses & Midwives – an agency of the Ghana Ministry of Health, but are available from the corresponding author on reasonable request.

## Appendix 1

**Table 7:** Methodology

Evaluation Objective 1: program outcomes										
						Evaluation objective 2: reintegration into the health system			Evaluation objective 3: satisfaction with training	
Cohorts	Knowledge Pre-Test & Confidence Survey (5-point Likert scale)		Objective Structured Clinical Examination (5-point scale)			Focus Groups		Reintegration Survey	Course Evaluation Surveys (5-point Likert scale)	Focus Groups
	Baseline	Graduation	Baseline	Graduation	14 months post-graduation	Graduation	14 months post-graduation	14 months post-graduation	Graduation	Graduation
Accra C1	X	X	X	X	X	✓	X	✓	✓	✓
Tamale C1	✓	✓	X	✓	X	✓	X	✓	✓	✓
Kumasi C1	✓	✓	X	✓	✓	✓	✓	✓	✓	✓
Accra C2	✓	✓	X	✓	✓	✓	✓	✓	✓	✓
Tamale C2	✓	✓	X	✓	✓	✓	✓	✓	✓	✓
Kumasi C2	✓	✓	✓	✓	NYA	X	NYA	NYA	✓	X
Accra C3	✓	✓	X	✓	NYA	✓	NYA	NYA	✓	✓
Tamale C3	✓	✓	X	✓	NYA	✓	NYA	NYA	✓	✓

X data was not collected or included

✓ Data is included in the analysis

*NYA* data not yet available

## Appendix 2

**Table 8:** Categories of competence used in OSCE rating scale and their definitions. Adopted from Bondy’s scale [18]

The following criteria describe the standard of behaviour or procedure, quality of Performance and level of Assistance required	
ND- Not Done	• Behaviour/ Procedure Not Done
1- Not competent	• Unsafe behaviour/ procedure • Unskilled • Required continuous cues^a^
2- Towards competent	• Requires guidance to ensure safety, Not completely accurate • The procedure does not accomplish the intended outcome • Unskilled, Inefficient, excess time and energy • Required frequent cues^a^
3- Competent	• Safe, Accurate, Intended outcome mostly achieved • Skilled, Hesitant, some excess time or energy noted • Required occasional supportive comments^b^
4- Towards proficient	• Safe, Accurate, Achieved intended outcome • Proficient, Minimal hesitation, good pace • Requires minimal supportive comments^b^
5- Proficient	• Safe, Accurate, Achieved intended outcome • Proficient, Confident, Efficient speed and style • No supporting comments required

^a^Supportive Cues involve providing the student with directions or prompts to guide their completion of the behaviour or procedure

^b^Supportive Comments involve statements of encouragement or agreement with the student’s behaviour or completion of the procedure

## Appendix 3

**Table 9:** Demographics of focus group discussion participants at graduation. 55 students from seven cohorts participated

Characteristic	N (%)
Years of experience as a nurse **Mean (SD)**	5.7 ± 3.4
**Gender**	
Female	43 (78)
Male	12 (22)
**Agency**	
Ghana Health Service	41 (75)
Ministry of Health	4 (7)
Christian Health Association	8 (15)
Quasi facility^a^	2 (4)
**Type of facility**	
Teaching Hospital	4 (7)
Regional Hospital	7 (13)
District Hospital	41 (75)
Polyclinic	1 (2)
Health Centre	1 (2)
CHPS^b^	1 (2)

^a^Quasi facility refers to the Police, Military, Prisons, public Technical/University and Mining hospitals and clinics

^b^*CHPS* community-based health planning and services

## Appendix 4

**Table 10:** Demographics of focus group discussion with graduates 14 months after graduation. Thirty-eight graduates from three cohorts participated

Characteristic	N (%)
Years of experience as a nurse **Mean (SD)**	4.0 ±1.3
**Gender**	
Female	29 (76)
Male	9 (24)
**Agency**	
Ghana Health Service	28 (74)
Ministry of Health	3 (8)
Christian Health Association	7 (18)
**Type of facility**	
Teaching Hospital	3 (8)
Regional Hospital	6 (16)
District Hospital	25 (66)
Polyclinic	1 (3)
Health Centre	2 (4)
CHPS ^1^	1 (3)

*CHPS* community-based health planning and services

## Abbreviations

MOH: Ghana Ministry of Health
OSCE: Objective Structured Clinical Examination
PNEP: SickKids-Ghana Pediatric Nursing Education Partnership
SDG: Sustainable Development Goal
