# Supplementary material for: Scaling up pediatric nurse specialist education in Ghana – a longitudinal, mixed methods evaluation
Source: BMC Nurs. 2021 Feb 16;20:32. doi: 10.1186/s12912-021-00550-1 (PMC7885484; doi:10.1186/s12912-021-00550-1)
Supplement: Supplementary file 1 — Additional file 1. [file 12912_2021_550_MOESM1_ESM.pdf]

Focus Group Discussion Guide with Residents

This tool is meant to help us measure:

- Confidence
- Knowledge
- Clinical skills
- Attitude
- Leadership
- Gender sensitivity

Conducted with: Students (Associate Nurses)
Focus Group Discussion date and time:
Facilitator names:
Note taker:
Location:

Script:
Introductions
Ground Rules for the focus group
Explaining the consent forms/demographic forms
Give an opportunity to participants to ask questions

Question	Measures
1. Could you please tell me what you liked most and least (or disliked) about the programme?	Opening question related to overall effectiveness/experience
2. Please give us a few examples of the most important skills that you have learned in this program that you intend to incorporate into your practice?	Change in practice broadly (application of knowledge & skills)
3. Results from other student cohorts to date show that overall GCNM Associate Resident graduates have increased confidence in their abilities after taking this program. In your view, what will be the impact of this increased confidence on your practice?	Application of confidence
4. Will you do anything differently in your practice now as the result of learning about gender sensitivity in the PNEP programme?	Gender-sensitivity/equity
5. Has taking part in the PNEP in any way influenced your attitude towards:<br>a) your patients?<br>b) your colleagues?<br>c) your profession?	Attitude
6. What types of leadership roles or activities do you hope to engage in when you go back to your workplace? (examples: development of policies and procedures, advocacy, or change projects to improve child health)	leadership
7. What more needs to be done to make the programme more beneficial to children and families in Ghana? (areas for improvement)	Overall programme effectiveness
ONLY IF THERE IS TIME LEFT, ask: 8) One of the things the program asked you to rate yourself on the first day was how confident you feel "managing conflict between myself and another member of the health care team". Do you feel more prepared to manage conflict now or not? Please tell us a little bit more about this.	Inter-professional dynamic
ONLY IF THERE IS TIME LEFT, ask : 9) Now that you are going back to work, what are some of the things that will<br>a) help you to apply the skills you have acquired to your everyday work?<br>b) hinder your ability to apply your skills in your everyday work?	Context

Thank you very much for your participation.
