# Supplementary material for: Scaling up pediatric nurse specialist education in Ghana – a longitudinal, mixed methods evaluation
Source: BMC Nurs. 2021 Feb 16;20:32. doi: 10.1186/s12912-021-00550-1 (PMC7885484; doi:10.1186/s12912-021-00550-1)
Supplement: Supplementary file 2 — Additional file 2. [file 12912_2021_550_MOESM2_ESM.pdf]

Focus Group Questionnaire: 14-months follow-up

1. **Opening question: How did the OSCE go?**
   Probes:
   a) *Which OSCE station did you think was the easiest/hardest?*
   b) *Why?* [You can ask them to raise their hands as you call out station names)

2. How would you describe your nursing role since completing the programme?
   Probes:
   a) *Is it different? In what ways?*
   b) *Are you able to use the skills you acquired? Can you give us some examples?*
   c) *If you are not able to use your skills to the fullest extent, what is stopping you?*
   d) *Did you feel you were well received when you returned to your position?*

3) Have you been able to *transfer some of your knowledge to your peers?*
   Probes:
   a) *Can you give us examples?*
   b) *How was this knowledge sharing received?*

4. Compared to before you took this programme, has your satisfaction level with your nursing role changed?
   Probes:
   a) *Tell us why?*

5) What were the most valuable parts of the programme to you, and what do you wish you had learned more about/what do you think was missing?

Thank you so much for taking the time to talk to us!
